# Optimization of an Ischemic Retinopathy Mouse Model and the Consequences of Hypoxia in a Time-Dependent Manner

**DOI:** 10.3390/ijms25158008

**Published:** 2024-07-23

**Authors:** Inez Bosnyak, Nelli Farkas, Dorottya Molitor, Balazs Meresz, Evelin Patko, Tamas Atlasz, Alexandra Vaczy, Dora Reglodi

**Affiliations:** 1 Department of Anatomy, HUN-REN-PTE PACAP Research Team, Medical School, University of Pecs, 7624 Pecs, Hungary; bosnyakinez99@gmail.com (I.B.); molittty@gmail.com (D.M.); mereszbalazs@gmail.com (B.M.); evelin.patko@gmail.com (E.P.); attam@gamma.ttk.pte.hu (T.A.); 2 Institute of Bioanalysis, Medical School, University of Pecs, 7624 Pecs, Hungary; nelli.farkas@aok.pte.hu; 3 Department of Sportbiology, Faculty of Sciences, University of Pecs, 7624 Pecs, Hungary

**Keywords:** retina, hypoxia, ischemia

## Abstract

The retina is one of the highest metabolically active tissues with a high oxygen consumption, so insufficient blood supply leads to visual impairment. The incidence of related conditions is increasing; however, no effective treatment without side effects is available. Furthermore, the pathomechanism of these diseases is not fully understood. Our aim was to develop an optimal ischemic retinopathy mouse model to investigate the retinal damage in a time-dependent manner. Retinal ischemia was induced by bilateral common carotid artery occlusion (BCCAO) for 10, 13, 15 or 20 min, or by right permanent unilateral common carotid artery occlusion (UCCAO). Optical coherence tomography was used to follow the changes in retinal thickness 3, 7, 14, 21 and 28 days after surgery. The number of ganglion cells was evaluated in the central and peripheral regions on whole-mount retina preparations. Expression of glial fibrillary acidic protein (GFAP) was analyzed with immunohistochemistry and Western blot. Retinal degeneration and ganglion cell loss was observed in multiple groups. Our results suggest that the 20 min BCCAO is a good model to investigate the consequences of ischemia and reperfusion in the retina in a time-dependent manner, while the UCCAO causes more severe damage in a short time, so it can be used for testing new drugs.

## 1. Introduction

The retina, as a projection of the central nervous system, has one of the highest levels of oxygen consumption and metabolic rates among mammalian tissues [1]. Lack of oxygen supply and impaired metabolism play a key role in the pathophysiology of the most common vision-threatening diseases such as diabetic retinopathy, glaucoma, age-related macular degeneration and retinopathy of prematurity [2,3,4,5]. Despite the prevalence of these diseases, there are no non-invasive treatment options in clinical practice. Current therapeutic options are intravitreal anti-VEGF drugs or corticosteroids, laser photocoagulation and surgical procedures [6]. Based on a recent randomized, multicentric clinical trial, neither laser photocoagulation nor anti-VEGF intravitreal injection treatment in infants with retinopathy of prematurity achieved a 90% success rate. Serious adverse events occurred after both laser photocoagulation and anti-VEGF, in 13.3% (ocular) and 24.0% (systemic) of patients and in 7.9% (ocular) and 36.8% (systemic) of patients, respectively [7]. In addition, vitrectomy, which is the gold standard modality for proliferative diabetic retinopathy, may also have serious consequences [8,9].

Therefore, finding new therapeutic targets and drugs for hypoxia-caused retinopathies is a focus of research interest. However, the latest techniques are in the early experimental phase or are extremely expensive [8,10,11,12,13]. Despite the wide range of basic research articles, the pathomechanism of these diseases is not fully understood [12,14]. In addition, many questions remain unsolved about the oxygen metabolism and consequences of hypoxia in the retina.

To address these unresolved questions, researchers apply various disease-specific animal models in experimental practice. Different types of artery occlusions are widespread models to study the consequences of hypoxia in the retina [15,16,17,18]. The blood supply of the retina arrives from two systems. The outer part of the retina, consisting of retinal pigment epithelium, photoreceptors and outer nuclear and plexiform layers, is supplied by the choroid plexus, which arises from the ciliary arteries of the ophthalmic artery. The inner part of the retina includes the inner nuclear, inner plexiform, ganglion cell and nerve fiber layers. The central retinal artery, also a branch of the ophthalmic artery, supplies these parts. The outer parts contain no blood vessels, avoiding interference with light transmission, while the inner retinal vasculature forms multiple capillary plexus layers [19,20].

The ophthalmic artery originates from the internal carotid artery, which is a branch of the common carotid artery. That is why unilateral (UCCAO) or bilateral common carotid artery occlusion (BCCAO) is used to induce retinal hypoxia. However, the optimal type and the duration of occlusion remain unknown and vary between animal species, including different mouse strains [16,21,22,23].

According to the literature, the outer part of the retina is more sensitive to hypoxia, but contradictory results can be found [24]. However, inner retinal layers also undergo degeneration in severe hypoxia, due to compromised blood flow compensation [25]. Retinal ganglion cells (RGCs) are neurons in the inner part of the retina that process and transmit incoming visual information from the retina to the visual processing areas in the brain. About 18 types of RGCs have been defined in humans and primates with distinct characteristics and specific functions in the visual pathway [26]. Although it is well known that degradation of RGCs plays a crucial role in vision loss caused by ischemic retinopathies [27,28,29], their response to hypoxia may depend on their type, their location within the retina and the duration of hypoxia [30,31,32,33,34,35,36], so further research is needed in this field.

The aim of our study was to develop an optimal ischemic retinopathy mouse model with UCCAO or BCCAO for different durations and to investigate the consequences of hypoxia with morphological methods in a time-dependent manner.

## 2. Results

### 2.1. Optical Coherence Tomography (OCT)

**Figure 1:**
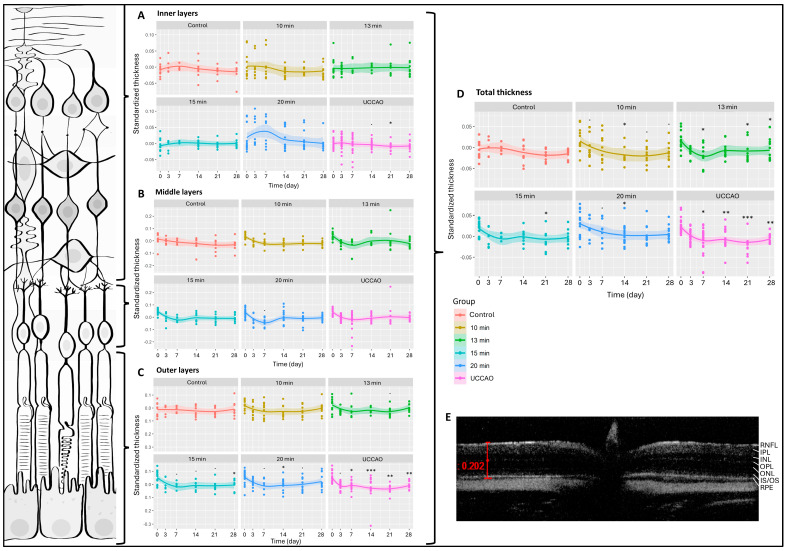
Optical coherence tomography (OCT) results and layers of the retina. The dots show the individual values, while the lines show the averages. Statistical analysis was performed with the linear random effect mixed model, and multiple comparisons were performed with Satterthwaite’s method. ˙ *p* < 0.1, * *p* < 0.05, ** *p* < 0.01, *** *p* < 0.001 vs. change in the control group. UCCAO: unilateral common carotid artery occlusion. Control: *n* = 10; 10-min BCCAO: *n* = 12; 13-min BCCAO: *n* = 14; 15-min BCCAO: *n* = 14; 20-min BCCAO: *n* = 17; UCCAO: *n* = 25. (**A**) OCT results of inner retinal layers. The upper bracket shows the inner layers of the retina. (**B**) OCT results of middle retinal layers. The middle bracket indicates the middle layers of the retina. (**C**) OCT results of the outer part of the retina. The bottom bracket shows the outer retinal layers. (**D**) OCT results of total retinal thickness. (**E**) A representative OCT image. Scale bar: 202 µm. RNFL: retinal nerve fiber layer. IPL: inner plexiform layer. INL: inner nuclear layer. OPL: outer plexiform layer. ONL: outer nuclear layer. IS/OS: inner and outer segments of photoreceptor cells. RPE: retinal pigment epithelium.

We had six measurement dates (days 0, 3, 7, 14, 21 and 28) and six groups (control; 10 min, 13 min, 15 min and 20 min BCCAO; and UCCAO). Altogether, 412 OCT measurements were performed during the experimental period and 5188 data were included in our analysis. The change in total retinal thickness was significantly greater after any degree of hypoxia compared to controls (Figure 1). This was most pronounced (*p* < 0.01) in the 20 min BCCAO and the UCCAO groups (Figure 1). After multiple comparisons, a significant reduction in retinal thickness was observed after 10 min of BCCAO on day 14 (*p* = 0.033), after 13 min of BCCAO on days 7, 21 and 28 (*p* = 0.047, 0.029 and 0.037, respectively), after 15 min of BCCAO on day 21 (*p* = 0.048), and after 20 min of BCCAO on day 14 (*p* = 0.040). UCCAO led to the most drastic thinning of the retina (day 7: *p* = 0.013; day 14: *p* = 0.007; day 21: *p* < 0.001; day 28: *p* = 0.006) (Figure 1).

Due to excessive degeneration of the retinal tissue, the software could not recognize the layers in the UCCAO group in some cases, so the detailed analysis contains 398 data per layer instead of 412. The inner retinal layers include the nerve fiber, inner plexiform and inner nuclear layers. The 20 min BCCAO group showed an overall significant change in the case of the inner layers of the retina (*p* = 0.016). However, one-by-one comparison was only significant on day 21 in the UCCAO group (*p* = 0.014) (Figure 1A). The middle retinal layers consist of the outer plexiform and the outer nuclear layers. Ischemia did not alter this part of the retina during the experimental period (Figure 1B). The outer part of the retina includes the inner and outer segments of the photoreceptors and the pigment epithelial layer. Based on our OCT measurements, these layers are prone to ischemia, because the linear mixed effect model revealed a significant change in the thickness of the outer retina in the 13 min BCCAO, 15 min BCCAO, 20 min BCCAO and UCCAO groups (*p* = 0.035, <0.001, <0.001, <0.01). A significant decrease was observed after 15 min of BCCAO on day 28 (*p* = 0.012), after 20 min of BCCAO on day 14 (*p* = 0.040) and after UCCAO on days 7, 14, 21 and 28 (*p* = 0.046, <0.001, <0.01, <0.01) (Figure 1C).

**Figure 2:**
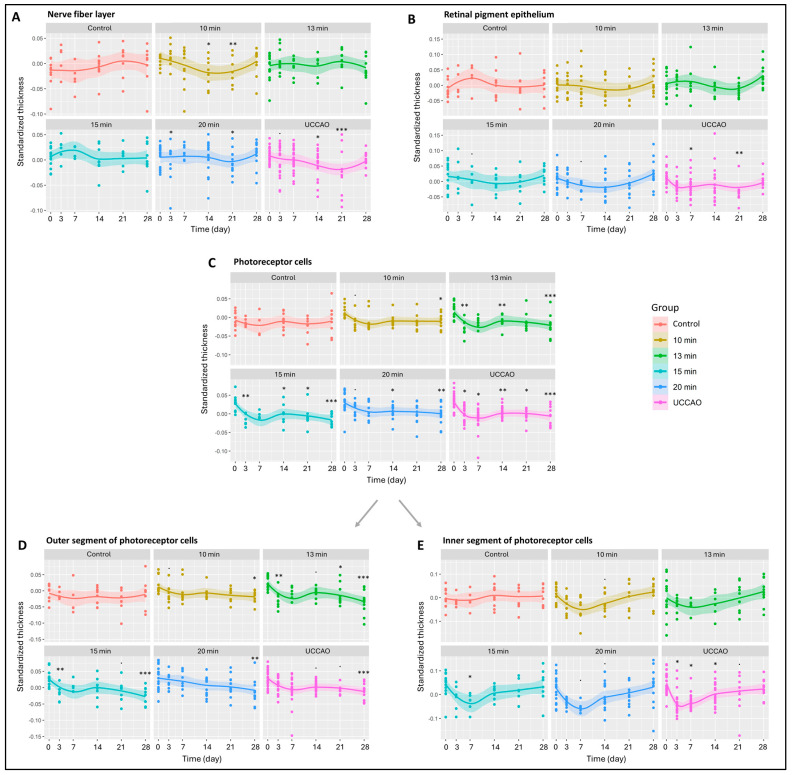
Results of optical coherence tomography (OCT) measurements of the different retinal layers. The dots show the individual values, while the lines show the averages. Statistical analysis was performed with the linear random effect mixed model, and multiple comparisons were performed with Satterthwaite’s method. ˙ *p* < 0.1, * *p* < 0.05, ** *p* < 0.01, *** *p* < 0.001 vs. change in control group. Control: *n* = 10; 10 min BCCAO: *n* = 12; 13 min BCCAO: *n* = 14; 15 min BCCAO: *n* = 14; 20 min BCCAO: *n* = 17; UCCAO: *n* = 25. The thickness of (**A**) nerve fiber layer; (**B**) retinal pigment epithelium; (**C**) whole photoreceptor cells; (**D**) outer segment of photoreceptor cells; (**E**) inner segment of photoreceptor cells.

All the retinal layers were also analyzed separately (Figure 2 and Figure S1) The nerve fiber layer, where the axons of ganglion cells are found, changed significantly in the 10 min, 15 min and 20 min BCCAO groups and in the UCCAO group (*p* = 0.034, 0.041, 0.016 and 0.011). UCCAO caused the greatest reduction in the nerve fiber layer (day 14: *p* = 0.019; day 21: *p* < 0.001). In addition to this, a notable change was observed in the 10 min (day 14: *p* = 0.027; day 21: *p* = 0.007) and 20 min BCCAO (day 3: *p* = 0.034; day 21: *p* = 0.023) groups (Figure 2A). After the innermost layer, the outermost layer was analyzed, where we found that the retinal pigment epithelium was significantly reduced in the UCCAO group by days 7 (*p* = 0.014) and 21 (*p* = 0.008) (Figure 2B).

Subsequently, the remaining layers of the retina were analyzed. The inner plexiform layer showed significant thinning only in the UCCAO group on day 21 (*p* = 0.033) (Figure S1). The inner nuclear layer (INL) consists of cell bodies of bipolar cells, Müller glial cells, amacrine cells, horizontal cells and, occasionally, displaced ganglion cells. A thickening tendency of the INL was observed in the 20 min BCCAO group by day 7. The INL thickness in this group was not significantly different from the initial value by the end of the experimental period. However, a significant thinning occurred in the UCCAO group by day 21 (*p* = 0.011) (Figure S1).

The outer plexiform layer was not markedly sensitive to ischemia; it changed significantly only in the 13 min BCCAO group on day 14 (*p* = 0.029) (Figure S1). Cell bodies of photoreceptors are found in the outer nuclear layer, which showed an overall significant change in the 13 min (*p* = 0.049) and 15 min (*p* = 0.035) BCCAO groups (Figure S1). A significant thinning was observed in the 20 min BCCAO group on day 7 (*p* = 0.038).

Moreover, OCT measurements showed a linear increase in degeneration with ischemia duration for the photoreceptor layer (10 min BCCAO: *p* = 0.029; 13 min BCCAO: *p* = 0.006; 15 min BCCAO: *p* < 0.001; 20 min BCCAO: *p* < 0.001; UCCAO: *p* < 0.001). A significant decline in the photoreceptor layer was observed after 10 min BCCAO on day 28 (*p* = 0.049), after 13 min BCCAO on days 3, 14 and 28 (*p* = 0.001, 0.010, <0.001), after 15 min BCCAO on days 3, 14, 21 and 28 (*p* = 0.005, 0.015, 0.043, <0.001), after 20 min BCCAO on days 14 and 28 (*p* = 0.021, 0.028) and after UCCAO on days 3, 7, 14, 21 and 28 (*p* = 0.014, 0.028, 0.002, 0.012, <0.001) (Figure 2C).

The photoreceptor layer includes the inner (IS) and outer segments (OS) of rods and cones. The OS of photoreceptors was prone to ischemia, as its thickness showed an overall reduction in the 13 min (*p* = 0.004), 15 min (*p* = 0.002) and 20 min BCCAO (*p* = 0.001) groups and also in the UCCAO group (*p* = 0.001) (Figure 2D). After a detailed analysis, we observed a significant degeneration in all ischemic groups (in 10 min on day 28 (*p* = 0.026), in the 13 min BCCAO group on days 3, 21 and 28 (*p* = 0.012, 0.044, <0.001), in the 15 min BCCAO group on days 3 and 28 (*p* = 0.017, <0.001), in the 20 min BCCAO group on day 28 (*p* = 0.008) and in the UCCAO group on day 28 (*p* < 0.001). The thickness of the IS changed significantly in the 15 min BCCAO (*p* = 0.028) and the UCCAO (*p* = 0.008) groups (Figure 2E). A decrease was observed after 15 min BCCAO on day 7 (*p* = 0.030) and after UCCAO on days 3, 7 and 14 (*p* = 0.011, 0.010, 0.016).

### 2.2. Retinal Ganglion Cells (Brn3a)

**Figure 3:**
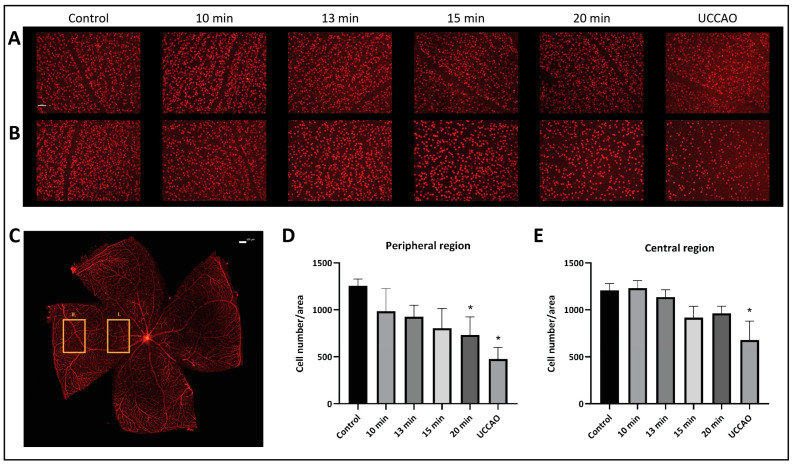
Brn3a labeling of retinal ganglion cells. Statistical analysis was performed with Kruskal–Wallis ANOVA and uncorrected Dunn’s post hoc test. *n* = 3/group. (**A**) Representative images of ganglion cells from the different groups in the central region of the retina under 20× magnification. Scale bar: 50 µm. (**B**) Representative images of ganglion cells from the different groups in the peripheral region of the retina under 20× magnification. Scale bar: 50 µm. (**C**) Representative image of a whole mount retina preparation. Rectangle I. indicates the location of the images of the central region, while the rectangle II. shows the location of the images of the peripheral region. Scale bar: 100 µm. (**D**) Ganglion cell count in the peripheral regions of the retina. * *p* < 0.05 vs. control. Area = 0.344 mm^2^. (**E**) Number of ganglion cells in the central regions of the retina. * *p* < 0.05 vs. control. Area = 0.344 mm^2^.

The number and the distribution of retinal ganglion cells were evaluated separately in the central and peripheral regions on retinal flat mounts (Figure 3). Our results show that the peripheral regions are more susceptible to ischemia. The mean (±SD) ganglion cell number per unit of area in the peripheral region was 1256 (±72) in the control group; 985 (±240) in the 10 min BCCAO, 926 (±124) in the 13 min BCCAO, 805 (±208) in the 15 min BCCAO, and 730 (±193) in the 20 min BCCAO group; and 477 (±124) in the UCCAO group (Figure 3A). A linear decreasing tendency could be observed with ischemia duration in the peripheral regions of the retina. The ganglion cell count was significantly lower in the 20 min BCCAO (*p* = 0.013) and the UCCAO (*p* = 0.001) groups compared to controls.

The mean (±SD) ganglion cell number per unit of area in the central region was 1207 (±75) in the control group; 1232 (±80) in the 10 min BCCAO, 1136 (±77) in the 13 min BCCAO, 917 (±120) in the 15 min BCCAO, and 961 (±77) in the 20 min BCCAO group; and 678 (±200) in the UCCAO group (Figure 3B). The ganglion cell count dropped significantly in the UCCAO group compared to controls (*p* = 0.006).

### 2.3. Retinal Stress (GFAP)

**Figure 4:**
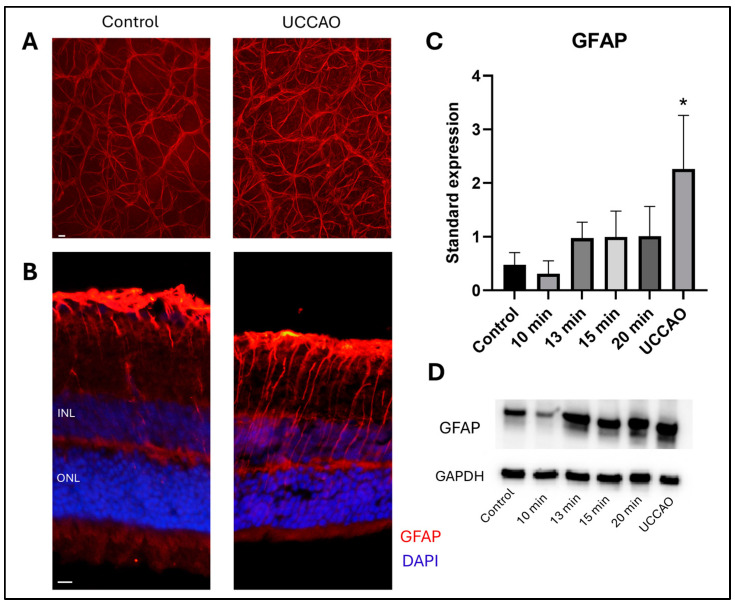
(**A**) Representative images of GFAP staining on retinal whole mounts from the peripheral regions of the retina. UCCAO: unilateral common carotid artery occlusion. Scale bar: 10 µm (**B**) Representative images of GFAP labeling with DAPI counterstaining on cross sections from the same groups as above. INL: inner nuclear layer. ONL: outer nuclear layer. Scale bar: 10 µm (**C**) Results of Western blot measurements. Statistical analysis: one-way ANOVA, Dunnet’s post hoc test. * *p* < 0.05 vs. control. *n* = 4/group × 3 runs (**D**) A representative blot of GFAP expression with the same groups as above. GFAP: glial fibrillary acidic protein. GAPDH: internal control.

Glial fibrillary acidic protein (GFAP) staining shows the reactive astrocytes and Müller glia cells in the retina [37]. Western blot analysis was performed to quantify the GFAP expression in the different groups (Figure 4D). Reactive gliosis was detected following retinal ischemia with overall one-way ANOVA analysis (*p* = 0.012) (Figure 4C). Furthermore, a significant increase in the standardized expression of GFAP was observed in the UCCAO group compared to controls (*p* = 0.006). In addition, an increasing tendency was found in the 13 min, 15 min and 20 min BCCAO groups compared to controls, but without statistical significance. In contrast, the GFAP expression was slightly, but not significantly, lower in the 10 min group than in the control group.

GFAP immunostaining was also performed to visualize the expression pattern in significantly different groups. Representative flat-mount images from the peripheral regions (Figure 4A) and cross-sections with nuclei counterstaining are shown (Figure 4). Locations of the central and peripheral regions are indicated in Figure 3C.

## 3. Discussion

In the present study, we described an optimized ischemic retinopathy mouse model for investigating the consequences of hypoxia in a time-dependent manner. BCCAO was performed for 10, 13, 15 or 20 min, followed by reperfusion. Furthermore, permanent UCCAO was also applied. Our results show that the 20 min BCCAO and the permanent UCCAO are promising models for studying the short- or long-term effects of ischemia in experimental practice.

Various types of artery occlusions are commonly used models to study hypoxia-induced degradation not only in the brain, but also in the retina. Permanent BCCAO in rats is a well-established and long-known method [18,38,39,40,41], but mice have numerous advantages. Although rats seem to be more complex and easier to handle in some situations, mice have been used as genetic models for over 100 years [42,43]. Knockout mice are more suitable and more readily available for investigating the role of different genes in these conditions. Numerous genes are specifically regulated in hypoxic conditions, as shown by transcriptomic analysis of the mouse retina after acute and chronic ischemia [44]. Regulation of these genes may contribute to structural changes in retinal tissue and its adaption to these situations [44]. Consequently, studies with knockout animals in this field could be of clinical relevance. We therefore aimed to compare the available mouse models of ischemic retinopathy and optimize one to study the consequences of hypoxia after oxygen deprivation of different durations.

Like in humans and rats, the blood supply of the mouse brain originates from the common carotid artery and from the vertebral artery. However, the connection between the vertebrobasilar and the carotid systems is uncomplete. For that reason, permanent BCCAO in mice causes severe global cerebral hypoxia and high mortality [45,46]. The presence or absence of the posterior communicating artery is a key question, but also, the terminology is not consistent across sources [21]. Unilateral common carotid artery occlusion and its disconnection is a promising possibility [22,47], but there are also drawbacks. We cannot rule out the hypoperfusion of the other side, so we did not use the contralateral eye as control. This was a limitation of our study. In addition, the damage was so severe in some cases that the software was unable to recognize the retinal layers separately. Another option is to reduce the blood supply by about 50% with micro-coils. However, a high degree of interindividual variability has been observed not only in inflammatory responses, but also in neuronal degeneration using this model [16].

Closing the blood supply of the retina directly with traditional surgical techniques [15] or with laser photocoagulation [48] mimics the characteristics of hypoxic conditions but has several unwanted consequences. Traditional surgery can cause severe destruction in the orbit, so the exact trigger of retinal degeneration could not be defined [15]. The effect of laser photocoagulation is unpredictable and perhaps temporary. Additionally, there is a chance of vascular perforation [48]. Intravascular injection of thrombin is also a potential method for artery occlusion [49]. Though these models can proxy for clinical situations, they are expensive and methodologically challenging under experimental conditions.

Ten minutes of BCCAO led to a slight reduction in retinal thickness in CD1-IGS mice based on routine histology [50]. A study showed that occluding one common carotid artery with a silk suture and the other with a microclip for 2 s can induce acute retinal ischemia [51]. Twenty minutes of BCCAO has also been used in mice but for purposes other than studying the retinal tissue [52]. Therefore, we performed BCCAO for different durations between 10 and 20 min.

Change in the thickness of retinal layers was monitored by OCT. This imaging technique is a painless method with high translational value because it is widely used in the clinical practice [53]. It also holds promise for the development of pathological diagnostic methods in other medical disciplines [54]. Unlike conventional histological methods, OCT measurements can be repeated. Therefore, we took OCT snapshots several times during the 1 month experimental period. The measurement dates were chosen based on the literature [55,56].

In contrast to a previous study [56], we observed a significant decrease in total retinal thickness after UCCAO on multiple days. In this study, no morphological changes were observed in spite of functional decline 3, 7 and 14 days after UCCAO [56]. In addition, we showed a decrease in total retinal thickness not only in UCCAO, but also in some BCCAO groups. The different, almost contradictory results found in the literature can be explained by the method used inducing ischemia, the age of the subjects, the different anesthetic compounds, the duration and severity of the hypoxia and the time points studied [47]. In addition, retinal edema caused by oxygen deprivation may occur but not in all types of hypoxic conditions [57].

The nerve fiber layer, where the axons of retinal ganglion cells are found, was sensitive to ischemia. Clinical studies underline the importance of the RNFL thickness as a biomarker in ischemic conditions [58]. RNFL thinning was reported after cerebral infarction [59] and in the early stage of diabetic retinopathy [60]. Cocaine causes tissue ischemia, and RNFL thinning was found in cocaine users compared to controls [61]. RNFL thickness can be evaluated fast and noninvasively, and it may also show the severity of the brain atrophy [61]. Interestingly, an initial thickening of the nerve fiber layer was detected three days after excitotoxic retinal injury [55]. A similar tendency was found in our 15 min BCCAO group, but without significance.

The thickness of the inner nuclear layer also showed an initial thinning in the 20 min BCCAO group. Although an article reported that INL is susceptible to mild ischemia [62], its thickness decreased significantly only in the UCCAO group. Moreover, functional loss of cells in the inner nuclear layer was detected by electroretinography [63]. Similar to previously published data [44], the outer nuclear layer did not show remarkable changes after ischemia. Sensitivity of the photoreceptor cells was also analyzed by measuring the thickness of the inner and outer segments. The thinning of the photoreceptor layer during the experimental period was significant in all ischemic groups compared to controls. Hypoxic conditions lead to a shortening of photoreceptors, and this damage easily becomes irreversible [44,64]. A previous study also highlights that cones are especially sensitive to ischemic-reperfusion injury [63]. This finding was confirmed by a clinical study that highlighted changes in the morphology and signal density of cones in diabetic retinopathy due to impaired capillary circulation [65]. We observed severe photoreceptor damage, but milder degeneration is not always visible with OCT [66], representing a limitation of these examinations.

The last layer, the thickness of which was measured by OCT, was the pigment epithelial layer. This layer changed remarkably after UCCAO in the experimental period. The retinal pigment epithelium consists of cuboidal cells that interact with photoreceptors and support their function. The degradation of pigment epithelial cells is soon followed by the atrophy of photoreceptors [67]. Functional impairment of these cells has a key role in the development of age-related macular degeneration [68]. In addition, ischemia followed by reperfusion causes the release of reactive oxygen species, which are mainly produced in the mitochondria. This mechanism can easily lead to pigment epithelial damage, due to the high mitochondria content of these metabolically active cells [69,70].

Pigment epithelium has an essential role in light transmission, but the retinal ganglion cells transmit visual information to the specific areas of the brain [71]. Immunolabeling of retinal ganglion cells was performed, because most SD-OCTs used in the experimental practice are not able to measure the thickness of the ganglion cell layer separately or these features are not sufficiently precise. Based on the literature, the ganglion cell complex (GCC) can help predict the loss of retinal ganglion cells [72]. The GCC includes the nerve fiber layer, the ganglion cell layer and the inner plexiform layer. However, the GCC can show the ganglion cell loss only if more than half of these cells have died [72]. Retinal ganglion cells were labeled with Brn3a, a commonly used antibody for detecting these neurons [73]. The ganglion cell number significantly decreased after ischemia and this was more severe in the peripheral regions and in the 20 min BCCAO and UCCAO groups. Based on an ex vivo study, RGCs lost their activity after hypoxia. The longer the duration of hypoxia, the less their activity returned [31]. The loss of retinal ganglion cells has been extensively studied and has been proven to play a crucial role in the development of visual impairment in glaucoma, diabetes and other optic neuropathies [74,75,76,77].

Moreover, the expression of GFAP was measured with Western blot analysis and visualized with immunohistochemistry. Numerous retinal damaging factors, including ischemia, cause elevated GFAP expression [63,78]. The GFAP expression was only significantly higher in the UCCAO group, but an increasing tendency was observed in the 13, 15, and 20 min BCCAO groups compared to the controls. Interestingly, the expression of GFAP after 10 min BCCAO did not even reach the level of the control group. A protective autoregulatory response after mild hypoxia may explain this observation [79]. Additionally, ischemic postconditioning can also have positive effects [80,81], so the severity and duration of ischemia are crucial in estimating the retinal consequences in hypoxic conditions.

In conclusion, we studied the consequences of different durations of hypoxia in a time-dependent manner, as impaired oxygen supply plays a key role in the pathophysiology of the most common vision-threatening diseases such as diabetic retinopathy, glaucoma, age-related macular degeneration and retinopathy of prematurity [2,3,4,5]. Based on our results, 20 min BCCAO is a good model for studying the effects of hypoxia in the long term and for examining the sensitivity of different cell types. Moreover, UCCAO causes more severe retinal tissue damage in CD1-IGS mice, so this method could be used to test potential new drugs.

## 4. Materials and Methods

### 4.1. Animals

Four-to-five-month-old, male CD1-IGS mice (*n* = 116) were used in this study. Animals were watered, fed ad libitum and maintained under a 12 h light/dark cycle. All procedures were undertaken in accordance with the Animal Research Review Committee of the University of Pecs, Hungary (BA02/2000-02/2022) and directives of the National Ethical Council for Animal Research, the European Communities Council (86/609/EEC), and ARVO Statement for the Use of Animals in Ophthalmic and Vision Research. Mice were divided randomly into the following experimental groups: bilateral common carotid occlusion (BCCAO) for 10 min (*n* = 16), for 13 min (*n* = 18), for 15 min (*n* = 18) or for 20 min (*n* = 21), permanent right unilateral common carotid artery occlusion (UCCAO) (*n* = 29) and sham-operated (*n* = 14), referred to as 10 min, 13 min, 15 min, 20 min, UCCAO and control, respectively.

### 4.2. Surgeries

**Figure 5:**
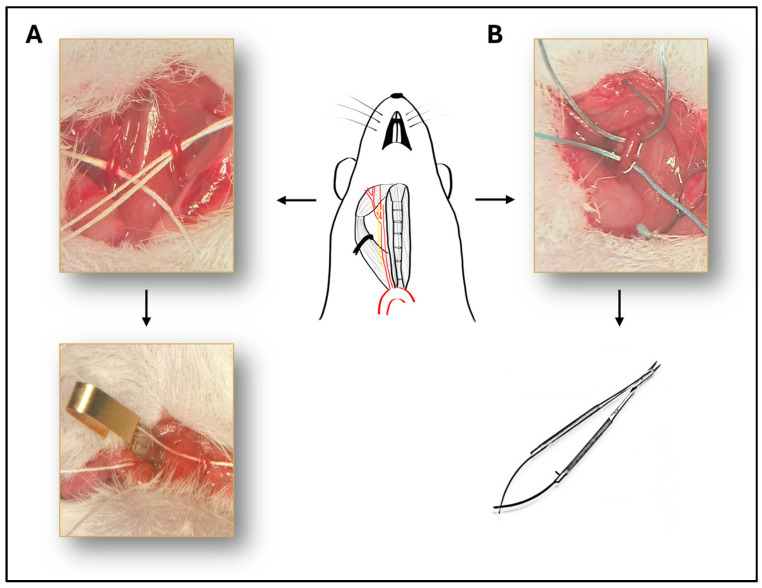
Illustration of the surgical procedure. Schematic drawing of the isolated right common carotid artery, with the vagus nerve next to it. (**A**) Representative picture of the bilateral common carotid artery occlusion (BCCAO). Both common carotid arteries are isolated and occluded with surgical microclips. (**B**) The right common carotid artery is occluded with two sutures and cut between the two sutures to avoid reperfusion.

Mice were anesthetized with intraperitoneal injection of ketamine (90 mg/kg, Calypsol, Richter Gedeon, Budapest, Hungary) and xylazine (10 mg/kg Sedaxylan, Dechra, Amsterdam, The Netherlands). Median neck incision was performed after topical disinfection with Braunol solution (B. Braun Medical AG, Sempach, Switzerland). Glands and muscles were pulled away and common carotid arteries were isolated with blunt dissection on the right side or on both sides depending on the experimental group (Figure 5). The vagus nerve was carefully separated from the artery during the dissection to avoid damaging the nerve. In case of BCCAO, both common carotid arteries were isolated and occluded with surgical microclips (Biemer-Clip, Aesculap, Melsungen, Germany) for 10, 13, 15 or 20 min followed by reperfusion (Figure 5A). In the case of UCCAO, the right common carotid artery was occluded with two sutures (3-0 non-absorbable sutures) and cut between the two sutures to avoid reperfusion (Figure 5B).

### 4.3. Optical Coherence Tomography (OCT)

Morphological analysis of the retinal layers was performed in vivo by spectral domain optical coherence tomography (Leica Microsystem, Bioptigen, Morrisville, NC, USA). Rectangular scans were taken with these parameters: 1000 A-scans/100 B scans × 3 Frames/each B-scan. A mouse retina imaging lens was used to take these scans, covering an area of 1.8 mm × 1.8 mm. OCT snapshots were taken under short-time general anesthesia with intraperitoneal ketamine (Calypsol, Richter Gedeon, Budapest, Hungary) and xylazine (Sedaxylan, Dechra, Amsterdam, The Netherlands) solution. Prior to the measurements, pupils were dilated with 0.01% atropine eye drops and artificial tears (Systane, Alcon, Budapest, Hungary) were used during the experiments to avoid damaging the corneal surface and to enhance image quality. OCT measurements were performed preoperatively (day 0) and 3, 7, 14, 21 and 28 days later in the following groups: control (*n* = 10), 10 min BCCAO (*n* = 12), 13 min BCCAO (*n* = 14), 15 min BCCAO (*n* = 14), 20 min BCCAO (*n* = 17) and UCCAO. Bioptigen InVivoVue Diver (v.3.3.7, Bioptigen Inc., Leica Microsystem, Durham, NC, USA) was used to analyze the thickness of the retinal layers. The auto-segmentation mode was used, so the software recognized the layers automatically.

### 4.4. Immunohistochemistry

Twenty-eight days after surgeries, mice (*n* = 3 × 3/group) were anesthetized with isoflurane and then cervical dislocation was performed. Eyeballs were carefully removed after cutting through the eyelids and the optic nerve. Eyes were dissected in 0.1 M phosphate-buffered saline (PBS). A small incision was made through the corneal limbus to increase the effectiveness of the fixation. Eyes were fixed in 4% paraformaldehyde dissolved in 0.1 M phosphate buffer for 1 h.

For whole mounts, retinas were then isolated in one piece after removal of the cornea and lens followed by washing six times for 5 min in 0.1 M phosphate-buffered saline (PBS). Retinas were blocked in 3% bovine serum albumin in PBS containing 4% normal donkey serum and 0.5% TritonX for 1 h at room temperature and incubated overnight at 4 °C with primary rabbit monoclonal antibody to Brn3a (Abcam, Cambridge, UK) diluted 1:100 or GFAP (G9269, Sigma-Aldrich, St. Louis, MO, USA) diluted 1:250 in the above-mentioned blocking solution. Samples were washed six times for 5 min in 0.1 M PBS at room temperature and then incubated with AlexaFluor 594 donkey anti-rabbit secondary antibody (Jackson ImmunoResearch, Ely, Cambridgeshire, UK) diluted 1:800. Four small cuts were made, and retinas were mounted on glass slides with Fluoroshield (SigmaAldrich, Budapest, Hungary) mounting medium. Ganglion cell number was evaluated under fluorescent light microscope (Nikon Eclipse 80i, Melville, NY, USA) under 20× magnification. Central and peripheral regions were analyzed separately (Figure 3).

For cross sections, eyecups were washed in 0.1 M PBS for 1 h following the fixation. Eyecups were then soaked in 10%, 20% and 30% sucrose solutions. We embedded the samples in O.C.T. compound-mounting medium (Tissue-Tek Cryo, Leica, Deer Park, IL, USA). Fourteen µm-thick sections were made with cryostat (LeicaCM1950, BioMarker, Budapest, Hungary) on gelatin-coated slides. After rehydration with 0.1 M PBS, sections were blocked for 2 h in the blocking buffer described above. Then, slides were incubated overnight at 4 °C with GFAP (1:250, G9269, Sigma-Aldrich) antibody. Alexa Flour 594 donkey anti-rabbit (Jackson ImmunoResearch, Cambridgeshire, UK) was used as secondary antibody. Finally, sections were washed in 0.1 M PBS and mounted with mounting medium with DAPI. Microphotographs were taken with Nikon Eclipse 80i. For more precise analysis, the quality of images was equally enhanced using Adobe Photoshop CS6 (Adobe Systems, Inc., San Jose, CA, USA).

### 4.5. Western Blot

Retinas (*n* = 4/group) were removed 24 h after the surgeries. Retinal homogenates were prepared as described earlier [82]. Bradford reagents were used for to measure the protein concentrations. Samples were then diluted with Laemmli buffer (1:1) and boiled for 5 min at 95 °C. Proteins were separated with SDS-PAGE gel electrophoresis with protein loads of 11 µL/lane and then transferred onto a nitrocellulose membrane. EveryBlot Blocking Buffer (BioRad; Hercules, CA, USA) was used for blocking the membranes for 5 min at room temperature. Membranes were incubated with GFAP (1:1000, G9269, Sigma-Aldrich) and GAPDH primary antibodies overnight at 4 °C. GAPDH (1:20,000, 2118l, Cell Signaling Technology Inc., Danvers, MA, USA) was used as an internal control. Membranes were washed for 30 min in Tris-buffered saline (TBS, pH = 7.5) with 0.2% Tween, followed by incubation with the secondary antibody (Goat Anti-Rabbit IgG (H + L)-HRP Conjugate, BioRad, USA) in a 1:3000 dilution for 2 h at room temperature. After washing steps, Pierce ECL Western Blotting Substrate (Thermo Scientific, Carlsbad, CA, USA) was used to visualize the bands. Quantification was performed by ImageJ software (v.1.54g).

### 4.6. Statistical Analysis

After evaluating the thickness of the total retina; the inner, middle and outer layers; and all layers separately, standardization was performed as described elsewhere [83] to eliminate the distortive effect of different measurement rounds. Statistical analysis was performed with these standardized results. A linear random effect mixed model was applied to compare the thickness of the layers depending on the duration of the ischemia and the time since the surgeries. The random effect was the ID of mice. Multiple comparisons were performed using Satterthwaite’s method.

To compare the ganglion cell count in the different groups, Kruskal–Wallis ANOVA and uncorrected Dunn’s post hoc test were performed. In addition, one-way ANOVA and Dunnet’s post hoc test were used to analyze the Western blot results.

Results were considered significant when *p* < 0.05. Statistical analyses were performed with GraphPad (9.5) and R Statistical Software (v4.3.2; 2023). The analysis and graphs were produced with lme4 [84] and ggplot2 [85] packages.

## Data Availability

The original contributions presented in the study are included in the article/Supplementary Material, further inquiries can be directed to the corresponding authors.

## Supplementary Materials

The following supporting information can be downloaded at: https://www.mdpi.com/article/10.3390/ijms25158008/s1.
