# Supplementary material for: Optimization of an Ischemic Retinopathy Mouse Model and the Consequences of Hypoxia in a Time-Dependent Manner
Source: Int J Mol Sci. 2024 Jul 23;25(15):8008. doi: 10.3390/ijms25158008 (PMC11311598; doi:10.3390/ijms25158008)
Supplement: Supplementary file 1 [file ijms-25-08008-s001.zip › ijms-3080318-supplementary.pdf]

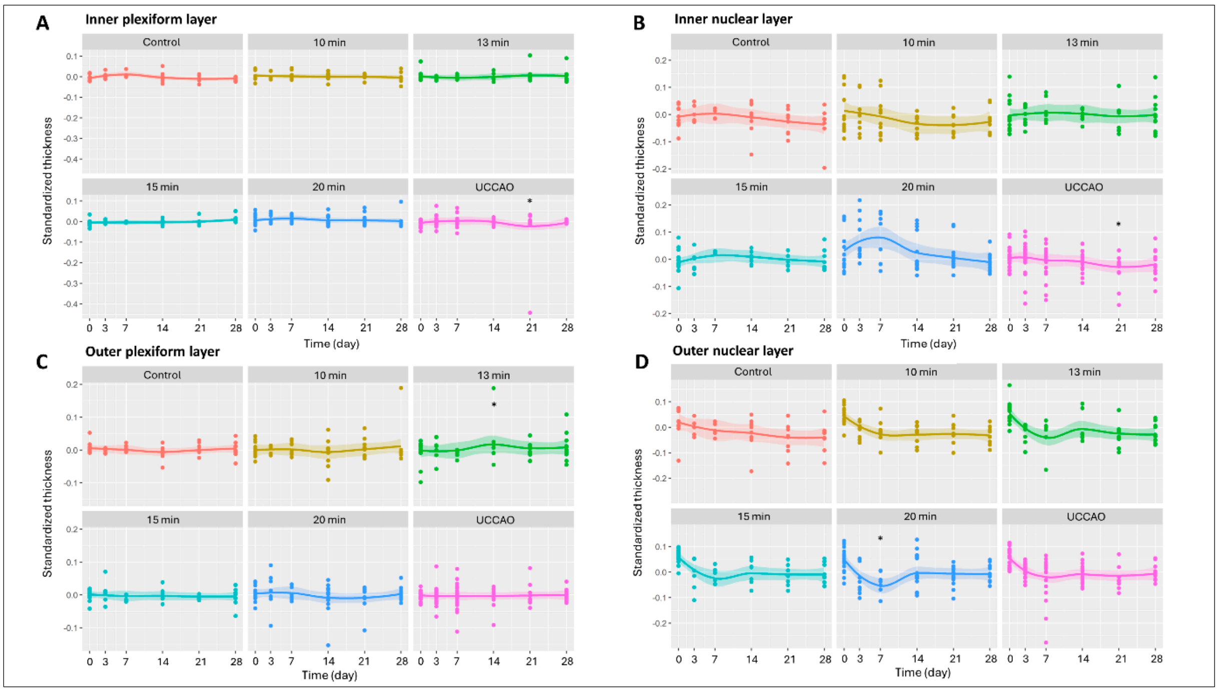

Figure S1. Results of optical coherence tomography (OCT) measurements of the different retinal layers. The dots show the individual values, while the lines show the averages. Statistical analysis was performed with the linear random effect mixed model, and multiple comparisons were performed with Satterthwaite's method. ˙$p<0.1$, *$p<0.05$vs. change in control group. A: inner plexiform layer. B: inner nuclear layer. C: outer plexiform layer. D: outer nuclear layer.
